# Age and Cohort Trends of Malignant Melanoma in the United States

**DOI:** 10.3390/cancers13153866

**Published:** 2021-07-31

**Authors:** Stephanie G. Lashway, Robin B. Harris, Leslie V. Farland, Mary Kay O’Rourke, Leslie K. Dennis

**Affiliations:** 1 Department of Epidemiology and Biostatistics, Mel and Enid Zuckerman College of Public Health, University of Arizona, Tucson, AZ 85724, USA; rbharris@arizona.edu (R.B.H.); lfarland@arizona.edu (L.V.F.); ldennis@arizona.edu (L.K.D.); 2 Department of Community, Environment and Policy, Mel and Enid Zuckerman College of Public Health, University of Arizona, Tucson, AZ 85724, USA; mkor@arizona.edu

**Keywords:** melanoma, epidemiology, SEER program

## Abstract

**Simple Summary:**

The occurrence of melanoma in the United States is increasing over time. We examined trends in melanoma by birth year and age groups to determine if individuals born more recently experience higher rates of melanoma as they age. We examined these trends separately among men and women and by the location on the body that the melanoma occurred. Melanoma incidence has continued to increase across more recent birth years and varies by body site and sex. Melanoma incidence will likely continue to increase as younger individuals age. While these are mostly thin melanomas, treatment to prevent cancer progression is still costly, both economically and emotionally, for patients.

**Abstract:**

The incidence of malignant melanoma in the United States is increasing, possibly due to changes in ultraviolet radiation (UVR) exposure due to lifestyle or increased awareness and diagnosis of melanoma. To determine if more recent birth cohorts experience higher rates of melanoma as they age, we examined age and birth cohort trends in the United States stratified by anatomic site and cancer type (in situ vs. malignant) of the melanoma diagnosed from 1975–2017. Poisson regression of cutaneous melanoma cases per population for 1975–2017 from the Surveillance, Epidemiology, and End Results (SEER) cancer registries was used to estimate age adjusted incidence for five-year birth cohorts restricted to Whites, ages 15–84. The rate of melanoma incidence across birth cohorts varies by anatomic site and sex. Melanomas at all anatomic sites continue to increase, except for head and neck melanomas in men. Much of the increase in malignant melanoma is driven by cases of thin (<1.5 mm) lesions. While increased skin exams may contribute to the increased incidence of in situ and thin melanoma observed across birth cohorts, the shifts in anatomic site of highest melanoma incidence across birth cohorts suggest changes in UVR exposure may also play a role.

## 1. Introduction

Cutaneous melanoma is the fifth most common cancer in the United States (US) [1]. As the deadliest of the three main types of skin cancer, melanoma mortality accounts for 1.1% of all cancer deaths in the US [1]. The incidence of malignant melanoma in the US is on the rise, with the overall age-adjusted incidence increasing over two-fold, from 10.5 per 100,000 in 1980 to 25.38 per 100,000 in 2017 [1,2]. The incidence of in situ melanoma increased an even greater amount from <1 per 100,000 in 1973 to 14 per 100,000 in 2006 [3]. Similar period trends are documented in Australia and Europe [4,5,6].

This increase is hypothesized to be due to changes over time in both skin screening practices and ultraviolet radiation (UVR) exposure [7,8,9]. Greater public awareness and more skin screenings by dermatologists have facilitated more diagnoses of melanomas, particularly for in situ and thin lesions [7,8]. Artificial UVR exposure from tanning devices has increased in North America and Europe over the last several decades [9,10,11]. Other changes to UVR exposure patterns may have occurred, including increased prevalence of intense intermittent exposures and changes in use of sun protection [11,12]. Prior research indicates that anatomic site of the melanoma lesion is correlated with the pattern of UVR exposure (chronic vs. intermittent) and varies by sex [13,14,15,16,17]. 

Despite the observed changes in melanoma incidence, few studies have been published on US melanoma trends by birth years (birth cohorts) in the last 30 years. Previous work reported that melanoma incidence within birth cohorts increased steeply through 1945 then began to stabilize [18,19]. However, their data were not recent, so it is unclear whether rates of melanoma leveled off for people born after 1950 [18,19]. SEER rates from 1974–1986 showed that melanoma birth cohort trends varied by anatomic site [18], although a more recent publication on international rates did not examine trends by anatomic site [19]. The increasing incidence of melanoma of the trunk diagnosed in young women starting in the 1980s is one noted pattern [15,20,21,22]. It is unclear whether this relatively high incidence in young women may affect rates as they age. Therefore, we sought to determine whether recent birth cohorts experience higher rates of melanoma than earlier birth cohorts and if the melanomas were thick or thin. We examined age and birth cohort trends of melanomas in the US diagnosed over the time-period of 1975–2017 stratified by anatomic site of the melanoma and by Breslow depth.

## 2. Materials and Methods

Incident cancer cases reported between 1975–2017 were analyzed from the National Cancer Institute’s Surveillance, Epidemiology, and End Results (SEER) program’s nine original population-based registries. The nine registries include the states of Connecticut, Hawaii, Iowa, New Mexico, and Utah, as well as the metropolitan areas of Detroit, Atlanta, San Francisco-Oakland, and Seattle-Puget Sound [23]. Cases were restricted to White individuals 15–84 years of age. Melanoma is rare in children less than 15 years of age [24]. For ages greater than 84 years (85–100+), birth year could not be estimated as the age category was too large. Consistent with SEER’s definition of incidence, our analyses include all primary cases of melanoma, regardless of whether an individual had a previous melanoma [1].

We restricted analysis to cutaneous melanoma as defined using the International Classification of Disease-Oncology (ICD-O) third edition. Histology codes 8720–8799 with behavior codes 2 (in situ) or 3 (malignant), and primary site codes C44.0–C44.7 were included, whereas cases of overlapping anatomic sites (C44.8), sites not otherwise specified (C44.9) and melanomas of the eyes and genitals (C69.0–C69.9) were excluded [25]. Anatomic site was categorized based on primary site codes as head and neck, trunk, arms, or legs. Five-year cohorts were constructed based on year of birth to examine birth cohort effects on melanoma incidence. Birth year was calculated by subtracting the age at diagnosis from the year of diagnosis for both cases and population data [23,26]. Due to small numbers of cases, birth years before 1900 and after 1989 were excluded. Age was categorized into 5-year age-groups. As SEER began collecting Breslow depth information for malignant melanomas in 1988, analysis of the incidence trend by Breslow depth was limited to cases diagnosed from 1988–2017, making the 1900–1904 birth cohort incomplete, and thus excluded from that analysis. 

Poisson regression was used to describe the probability of observing a number of cases per population in a fixed interval [27]. Poisson regression with an offset of the natural log of the population was conducted to estimate incidence (per 100,000 population) for each of the five-year birth cohorts. The 1940–1944 birth cohort and the 50–54 age-group were used as the reference categories for birth cohorts and age groups, respectively. We tested the assumption of Poisson regression that the variance of the incidence rate was equal to the mean of incidence rate [27,28]. The variance was larger than the mean incidence rate, indicating the model was over-dispersed [28]. Thus, a scale adjustment using a multiplicative overdispersion parameter of the deviance divided by the degrees of freedom was used to account for overdispersion [28]. Age and cohort-specific incidence was estimated from the Poisson model using least squared means. Regression was stratified by sex and anatomic site or Breslow depth. Analyses were conducted in SAS 9.4.

## 3. Results

Among the 168,488 cases of malignant melanoma and the 52,995 cases of in situ melanoma included in analysis, the majority were male (55.7% and 56.8%, respectively) and slightly over half (52.8% and 50.6%, respectively) were born after 1944 (Table 1). Cases among men were diagnosed at an older mean age than cases among women for both malignant and in situ melanoma, regardless of birth cohort. In men, the most common anatomic site of malignant melanoma was the trunk (41.8%), and the least common anatomic site was the legs (9.4%). In contrast, the legs were the most common anatomic site of malignant melanoma for women (32.3%). The least common anatomic site of malignant melanomas for women was the head and neck, while that site was the second most common among men (Table 1). The most common anatomic sites of in situ melanomas differed from those of malignant melanomas. In situ melanomas were most common on the head and neck among men and most common on the arms among women (Table 1). 

**Figure 1:**
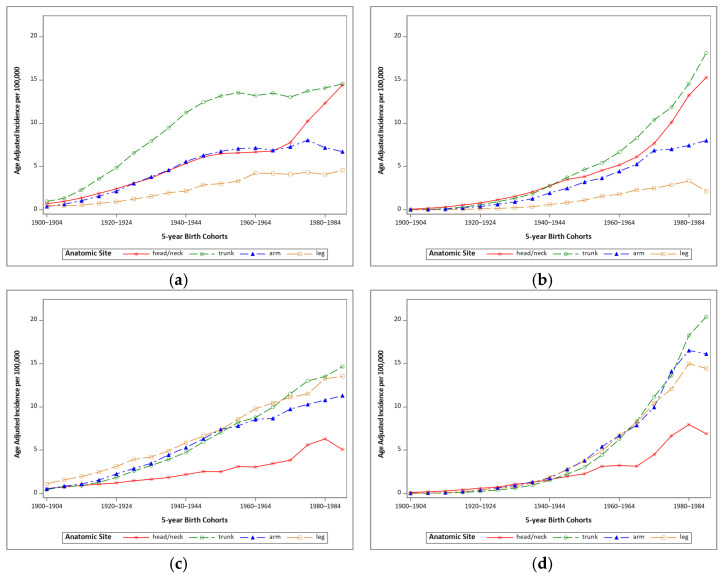
Age-adjusted incidence of melanoma for 5-year birth cohorts by anatomic site of original lesion. (**a**) malignant melanoma in males, (**b**) in situ melanoma in males, (**c**) malignant melanoma in females, (**d**) in situ melanoma in females. Data from SEER 9, 1975–2017.

Figure 1 illustrates the age-adjusted incidence of malignant and in situ melanoma for five-year birth cohorts from 1900 to 1989 for men and women stratified by anatomic site. For men, the anatomic site with the highest incidence of both malignant and in situ melanoma was the trunk (Figure 1a,b). For women, both malignant and in situ melanoma of the arms, legs, and trunk increased consistently from older to younger birth cohorts (Figure 1c,d). Malignant melanoma of the head and neck stayed consistently lower than other anatomic sites in women born from 1915 onwards, while for men it has increased steeply among those born after 1969 (Figure 1a,c).

**Figure 2:**
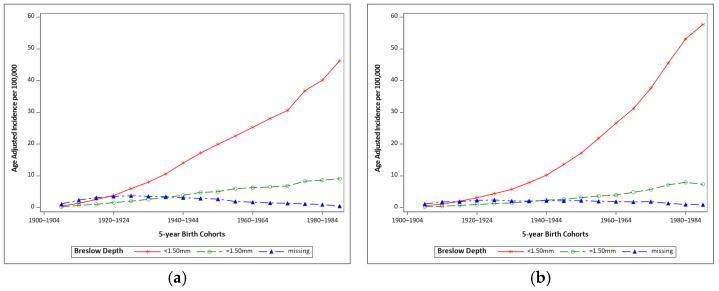
Age-adjusted incidence of malignant melanoma for 5-year birth cohorts by Breslow depth category for (**a**) males and (**b**) females. Data from SEER 9, 1975–2017.

Cases of malignant melanoma diagnosed from 1988–2017 (144,894 cases total) were included in the analysis of birth cohort trends by Breslow depth. Thickness trends were not analyzed for cases of in situ melanoma as 95.7% of in situ cases lacked a Breslow depth measurement. Across birth cohorts from 1905 to 1989, incidence of thin (<1.50 mm) malignant melanoma increased steeply, while thick (≥1.50 mm) increased slightly, but steadily (Figure 2). The amount missing Breslow depth measurements decreased across birth cohorts. The incidence of thin malignant melanomas is higher in women than men (Figure 2).

**Figure 3:**
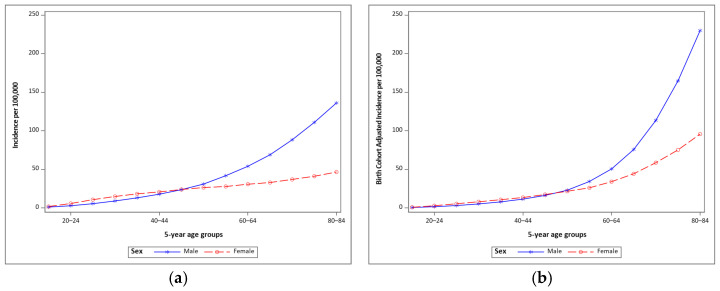
Incidence of malignant melanoma for 5-year age groups by sex, (**a**) crude, and (**b**) adjusted for 5-year birth cohort. Data from SEER 9, 1975–2017.

Malignant melanoma incidence increased with age for both men and women with higher rates among women under age 45 (Figure 3). After adjustment for birth cohorts the incidence at older ages dramatically increased (Figure 3).

**Figure 4:**
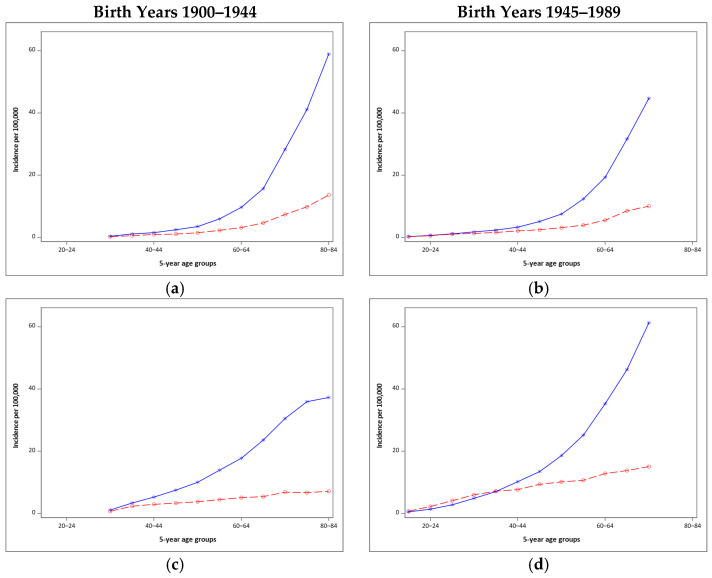

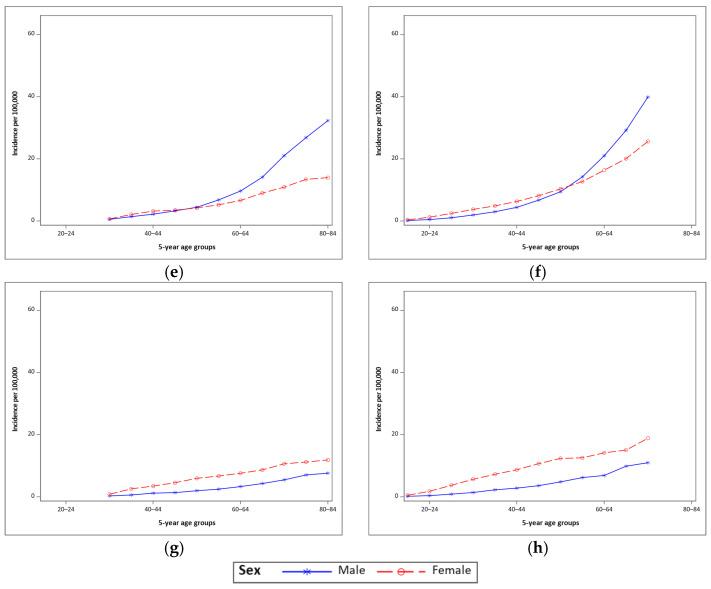
Incidence of malignant melanoma of four anatomic sites for 5-year age groups by sex and birth year. (**a**) head & neck birth years 1900–1944, (**b**) head & neck birth years 1945–1989, (**c**) trunk birth years 1900–1944, (**d**) trunk birth years 1945–1989, (**e**) arms birth years 1900–1944, (**f**) arms birth years 1945–1989, (**g**) legs birth years 1900–1944, (**h**) legs birth years 1945–1989. Data from SEER 9, 1975–2017.

Figure 4 shows the relationship of malignant melanoma with age varies by birth cohorts and anatomic site. Women have higher rates of melanoma of the leg than men regardless of age and birth cohort (Figure 4g,h). Among people born 1900–1944, men consistently had higher incidence of malignant melanoma of the trunk than women at all ages (Figure 4c). However, women, ages 20–39 years, born between 1945–1989 experienced higher rates of truncal melanoma than men (Figure 4d).

## 4. Discussion

These analyses show sharp increases in both in situ and malignant melanoma incidence with each subsequent birth cohort through the mid-1940s; however, after 1945 our results show a somewhat different picture. For men born after 1945, the incidence of in situ melanoma continues to sharply increase across more recent birth cohorts, particularly in situ melanomas of the trunk and the head and neck, while the rate of increase in malignant melanoma is attenuated for all anatomic sites except the head and neck (Figure 1b,c). The trends suggest that incidence of malignant head and neck melanomas in men may become equivalent to those of trunk melanomas (the last cohort within each analysis should not be over interpreted). In contrast, rates of both malignant and in situ melanoma for women continue to increase for recent birth cohorts at all anatomic sites (Figure 1c,d). Much of the increase in malignant melanoma appears attributable to thin rather than thick melanomas. 

Our updated results of prior SEER analyses show sharper increases after 1945 birth cohorts for both men and women than the prior report [18]. These findings are consistent with Bradford et al.’s report in 2010 of a low longitudinal age trend and high net drift for female truncal melanoma, which indicated melanomas of the trunk were increasing in young women across time [20]. Similarly, our results are consistent with international trends of melanoma that show increase over time and with each subsequent birth cohort [19]. The sharp increase in thin melanoma across birth cohorts is consistent with reported time-period trends of Breslow depth showing that thick tumors increased, but at lower rates than thin tumors [29].

Over the last century, patterns of UVR exposure have changed, as have clothing choices for both men and women [11]. The prevalence of reported sunburns has increased among US adults between 1986 and 1996, 1999 and 2004, and 2005 to 2015 [30,31,32]. Another study reported that the prevalence of sunburns did not decrease among adolescents and their parents between 1998 and 2004, even though sunscreen use increased [12,33]. In contrast to sunburn prevalence rates in the US, a study also found that the frequency of sunburns in birth cohorts relative to 1910–1919 only increased with each subsequent birth cohort until 1950–1959, which may indicate that either secular trends in sunburn prevalence do not reflect cohort trends or that international trends do not reflect trends specific to the US [34]. The study did find increased tanning bed use with each subsequent birth cohort [34], which could reflect tanning attitudes. Starting in the 1920s, tanned skin began to become desirable and led to the use of artificial UVR (tanning beds) in the late 1970s [11]. Regardless of whether the exposure is via natural sunlight or artificial UVR, the body perceptions favoring tanning persist. Over 50% of adolescents report positive attitudes towards tanned skin and research shows that college aged women in the US continue to tan despite awareness of the risk of skin cancer [33,35,36,37].

The higher rates of malignant melanoma for women under the age of 45 compared to men seen in both the crude and birth cohort adjusted incidence may reflect both the importance of a tan among young women and their use of artificial UVR at higher rates than men [10,38,39]. The differences between men and women appear to be the strongest in more recent cohorts for the legs and arms, with slightly higher rates until age 30 for the trunk (Figure 4d,f,h). In earlier cohorts, women consistently had lower rates of melanoma of the trunk then men at all ages (Figure 4c). Incidence of malignant melanoma of the trunk began to surpass that of malignant melanoma of the arm in women born after 1954. For women born after 1969, the trunk became the anatomic site of highest malignant melanoma incidence. Researchers hypothesize increased incidence of melanoma of the trunk in women is due to artificial tanning, although artificial UVR increases risk of melanoma of all exposed skin [20,38,40]. Use of tanning beds is a well-documented melanoma risk factor, particularly for women younger than 40 years and the majority of tanning bed users are women [10,38,40]. The use of tanning beds increased among adolescents and adults through the early 2000s [10,12,30]. Tanning bed use may also be a source of intense intermittent UVR exposure, as some tanning beds emit higher levels of UVR than the natural mid-day sun of Southern Europe [10]. Changes in the relative incidence of melanoma at specific anatomic sites may indicate differences in patterns of UVR exposure across birth cohorts, as melanomas at different anatomic sites are associated with particular patterns of sun exposure [17,41]. Melanomas of the trunk are thought to be associated with intermittent patterns of UVR exposure [17,41]. Our data on the increased rates of melanomas of the trunk in women born since 1944 are consistent with the hypothesis that increased tanning bed use over time increased melanoma incidence.

Both UVR exposure behaviors and biological differences may contribute to differences in melanoma incidence between men and women. As discussed previously, women intentionally tan (via sunbathing or artificial UVR) more than men [36,42,43]. However, research has also shown that women tend to use sunscreen more frequently than men [42,43]. One hypothesis is that the overall higher rate of melanoma in males is due to hormonal and immunological differences which may put females at slightly lower risk of developing melanoma, although it is not clear [20,44,45,46]. Nevi (associated with increased melanoma risk) have been found to be more prevalent in female than male college students [47]. According to the dual pathway hypothesis, melanoma occurrence at younger ages is more driven by nevi proneness and intermittent sun exposure than chronic sun exposure [48,49,50]. Our results show that the age at which men began to experience higher malignant melanoma incidence than women varied by birth cohort and anatomic site (Figure 4), which is consistent with the hypothesis that women’s UVR exposure patterns for recent birth cohorts has increased their risk at younger ages.

Additional factors that may contribute to increased melanoma incidence across birth cohorts are changes in melanoma awareness, screening, and diagnosis. Increased detection might explain the sharp increase in in situ and thin malignant melanomas seen across birth cohorts. Public awareness of melanoma has increased since the American Academy of Dermatology began sponsoring skin cancer screenings in 1985 [8]. Prior to the development and dissemination of the ABCD criteria for self-screening to the public in the 1980s, melanomas were diagnosed by clinically macroscopic features and more often at an advanced stage when they appeared large and/or ulcerated [7,41]. Technological advances for evaluating skin lesions, such as high-resolution dermoscopy, may play a role in the increased detection of in situ and invasive melanomas [7,41]. From the late 1980s to the late 2000s, research suggests a shift away from classifying a suspicious lesion as a dysplastic nevus towards classification as a thin melanoma [51].

While we lack direct exposure data, it is possible that changes in public awareness for screening, melanoma diagnosis strategies, and UVR exposure patterns all have contributed to the observed increase in malignant head and neck melanomas in birth cohorts of men born since 1965. Individuals may be more likely to observe changes to the skin of the face and neck than changes to the trunk or other sites; potentially leading to earlier and/or more frequent diagnoses of head and neck melanoma relative to other anatomic sites if combined with the documented lower diagnostic thresholds [7,51]. Prior research has suggested that men have higher melanoma incidence of the head and neck (sites chronically exposed to solar UVR) than other anatomic sites when adjusted for surface area [52]. Thus, changes in men’s fashion, both hats and, possibly, hairstyles, may have led to an increase in both intensity and duration of UVR exposure to the head and neck areas relative to cohorts born in the early 1900s. The decline of men’s hats began in the 1950s, accelerated in the 1960s, and by the 1970s hats ceased to be a typical part of everyday men’s wear [53]. While hats play a role in reducing UVR exposure and aiding melanoma prevention, hair also likely confers some protection against UVR and may reduce the amount of UVR to the skin by 81% [54,55]. Between the decreased use of sun protective clothing and changes in sun exposure behaviors, it is possible that the head and neck areas received more sun exposure relative to the trunk in cohorts of men born since 1965.

The findings from this study in the US suggest that the risk of an individual developing melanoma is greater for more recent birth cohorts, and that the annual burden of melanoma will increase as recent cohorts age. The increase is sharpest for in situ melanomas and thin malignant melanomas. It is of note, however, that similar birth cohort trends are not true in Australia where there has been a concentrated effort over decades to reduce UVR exposure through promotion of sun protection habits. Researchers in Queensland noted that while in situ melanoma increased, invasive melanoma incidence was decreasing for more recent cohorts [56]. They attributed this decrease, particularly for invasive melanoma diagnosed in persons younger than age 60, to their sun protection campaigns beginning in the 1980s [56]. Policies implemented in Australia to promote sun protection and reduce skin cancer incidence include: hat wearing during outdoor time at childcare centers and schools, grant programs for community shade construction, and sun protection items as tax deductible expenses for outdoor workers [57].

Strengths of these analyses include the use of population-based SEER national cancer data over four decades and examining incidence of melanoma by anatomic site. Examining malignant and in situ melanomas separately, as well as birth cohort trends by Breslow depth allowed for a more nuanced picture. The primary limitations of this analysis are that the registry data did not include any information on UVR exposures, and birth cohort trends could not be simultaneously adjusted for age and diagnosis time-period due to the correlation between the three. While SEER is a high-quality registry, the data does have limitations. It is a limitation that we were not able to exclude second melanomas due to lack of unique IDs between SEER locations and recent changes in SEER public access data to not include SEER location. Since SEER data are only available since 1975, it is also a limitation that each birth cohort does not contain the entire range of ages, which may affect the rates for the earliest and most recent cohorts. Further research directly comparing trends in UVR exposure factors and trends in melanoma incidence across birth cohorts could help clarify how much of the increase is due to UVR exposure. More importantly, given that in situ and both thin and thick malignant melanoma increased across birth cohorts, implementation of programs for behavior changes, similar to Australia’s programs, need to be developed to help decrease melanoma incidence in the US.

## 5. Conclusions

Both malignant and in situ melanoma incidence has continued to increase across birth cohorts with rates of increase varying by anatomic site and sex, and in situ more sharply increasing. While the vast majority of the increase in malignant melanomas across birth cohorts have been thin, thick melanomas have also increased. Changes in rates of melanoma in women do appear to parallel known changes in use of artificial tanning devises. Malignant melanoma incidence will likely continue to increase as younger birth cohorts age. Implementation of policy changes to increase prevention programs, such as done in Australia, is needed to decrease the incidence of melanoma in the US.

## Figures and Tables

**Table 1:** Cases of melanoma among White individuals in the United States born from 1900 through 1989 aged 15–84 years. Data from SEER 9, 1975–2017.

	Malignant Melanomas			In Situ Melanomas		
	Male	Female	Total	Male	Female	Total
All	93,841 (55.7%)	74,647 (44.3%)	168,488	60,866 (56.8%)	46,295 (43.2%)	52,995
Birth Years 1900–1944	49,673 (62.5%)	29,871 (37.6%)	47.2%	34,193 (64.5%)	18,802 (35.5%)	49.5%
Age at Diagnosis *	68.3 (10.5)	66.5 (11.6)	67.6 (11.0)	71.8 (8.1)	70.8 (8.8)	71.5 (8.4)
Birth Years 1945–1989	44,168 (49.7%)	44,776 (50.3%)	52.8%	26,673 (49.2%)	27,493 (50.8%)	50.6%
Age at Diagnosis *	49.0 (12.3)	44.3 (12.5)	46.7 (12.6)	54.4 (10.7)	49.2 (12.0)	51.8 (13.5)
Anatomic site of melanoma						
Head and Neck	23,877 (25.4%)	9484 (12.7%)	33,361 (19.8%)	27,532 (45.2%)	11,581 (25.0%)	39,113 (36.5%)
Trunk	39,183 (41.8%)	20,015 (26.8%)	59,198 (35.1%)	17,373 (28.5%)	10,138 (21.9%)	27,511 (25.7%)
Arms	21,938 (23.4%)	21,008 (28.1%)	42,946 (25.5%)	12,926 (21.2%)	13,139 (28.4%)	26,065 (24.3%)
Legs	8843 (9.4%)	24,140 (32.3%)	32,983 (19.6%)	3035 (5.0%)	11,437 (24.7%)	14,472 (13.5%)

* means and standard deviation.

## Data Availability

The data presented in this study are openly available through https://seer.cancer.gov/data/ (accessed on 23 July 2020).
